# Coronavirus-Associated Coagulopathy: Lessons From SARS-CoV1 and MERS-CoV for the Current SARS-CoV2 Pandemic

**DOI:** 10.7759/cureus.11310

**Published:** 2020-11-03

**Authors:** Shalinder Singh, Ufara Zuwasti, Christopher Haas

**Affiliations:** 1 Internal Medicine, MedStar Union Memorial Hospital, Baltimore, USA

**Keywords:** covid-19, covid-19-associated coagulopathy, sars virus, sars-cov2

## Abstract

To date, several studies have suggested a severe acute respiratory syndrome coronavirus 2 (SARS-CoV2)-mediated hypercoagulability in the forms of pulmonary embolism, stroke, gangrene, “COVID toes,” as well as other acute thrombotic complications, warranting the use of systemic anticoagulation. Currently, there are no definitive recommendations as to the timing and dosing of prophylactic or therapeutic anticoagulation in coronavirus disease 2019 (COVID-19) patients. In this manuscript, we report a case of SARS-CoV2-mediated hypercoagulability and review the literature pertaining to the incidence and pathophysiology of coronavirus-mediated coagulopathies. A 64-year-old female, with a medical history of hypothyroidism and remote tobacco abuse, presented to the ED with fever and nonproductive cough. She had multiple negative SARS-CoV2 nasopharyngeal PCR tests during her hospital stay, but chest imaging and elevated inflammatory markers were suggestive of SARS-CoV2 infection. Computed tomography showed a left upper lobe pulmonary embolism with associated right heart strain, and an enlargement of the main pulmonary artery, for which she was initiated on therapeutic anticoagulation with low molecular weight heparin. Despite the medical management of her pulmonary embolism and conservative management of her SARS-CoV2, her clinical condition worsened requiring intubation and mechanical ventilation. After seven days, she was successfully extubated and was transferred to the medical service where her clinical course remained stable and subsequently discharged home on apixaban. In patients with SARS-CoV1-, SARS-CoV2-, and the Middle East respiratory syndrome coronavirus (MERS-CoV)-mediated hypercoagulability, the risk of thrombosis appears to be multifactorial - direct viral cytopathological effects, a pro-inflammatory state, cytokine storm, hypoxia-inducible thrombosis, and endothelial inflammation culminating in the formation of intra-alveolar or systemic fibrin clots. While initial guidelines have been developed to assist clinicians in selecting appropriate chemoprophylaxis as well as therapeutic anticoagulation, a consensus statement remains lacking. Further studies are needed to evaluate the pathogenesis and treatment of coronavirus-induced thrombosis.

## Introduction

Clinically significant coronaviruses have long caused mild upper respiratory tract symptoms consistent with the common cold. In 2003 and 2013, however, two novel and more severe zoonotic coronaviruses, severe acute respiratory syndrome coronavirus (SARS-CoV1) and the Middle East respiratory syndrome coronavirus (MERS-CoV) emerged, with the capacity to cause significant morbidity and mortality, a feature not previously appreciated in coronavirus infection. Severe acute respiratory syndrome coronavirus 2 (SARS-CoV2) subsequently emerged in late 2019 as the third exceedingly pathogenic coronavirus and rapidly escalated into a highly communicable pandemic. As of September 25, more than 32 million people have been infected by SARS-CoV2 worldwide, with more than 980,000 deaths globally, though this likely represents a gross underestimation of the true number of cases [1]. Patients with SARS-CoV2 infection mainly present with respiratory symptoms, with a mild to the severe spectrum of illness, with more severe disease closely associated with dysregulated and excessive immune responses, termed a “cytokine storm.” Furthermore, there exist several asymptomatic cases as well as a wide variety of atypical presentations and additional clinical sequelae such as acute renal injury, myopericarditis, pulmonary embolism, and stroke [2]. As the pandemic has evolved, pulmonary embolism, stroke, gangrene, “COVID toes,” and other acute thrombotic complications have been increasingly recognized. To date, the SARS-CoV2 mediated hypercoagulable state remains incompletely understood but appears to portend increased morbidity and mortality. This hypercoagulable state has been labeled thrombo-inflammation, given its association with the underlying inflammatory state induced by SARS-CoV2, or alternatively Coronavirus Disease 2019 (COVID-19)-associated coagulopathy (CAC), which appear to be a distinct disease entity compared to other hypercoagulable states such as disseminated intravascular coagulation (DIC). In this article, we present a case of SARS-CoV2-mediated hypercoagulability and furthermore review the literature pertaining to the incidence and pathophysiology of coronavirus-mediated coagulopathies.

## Case presentation

**Figure 1:**
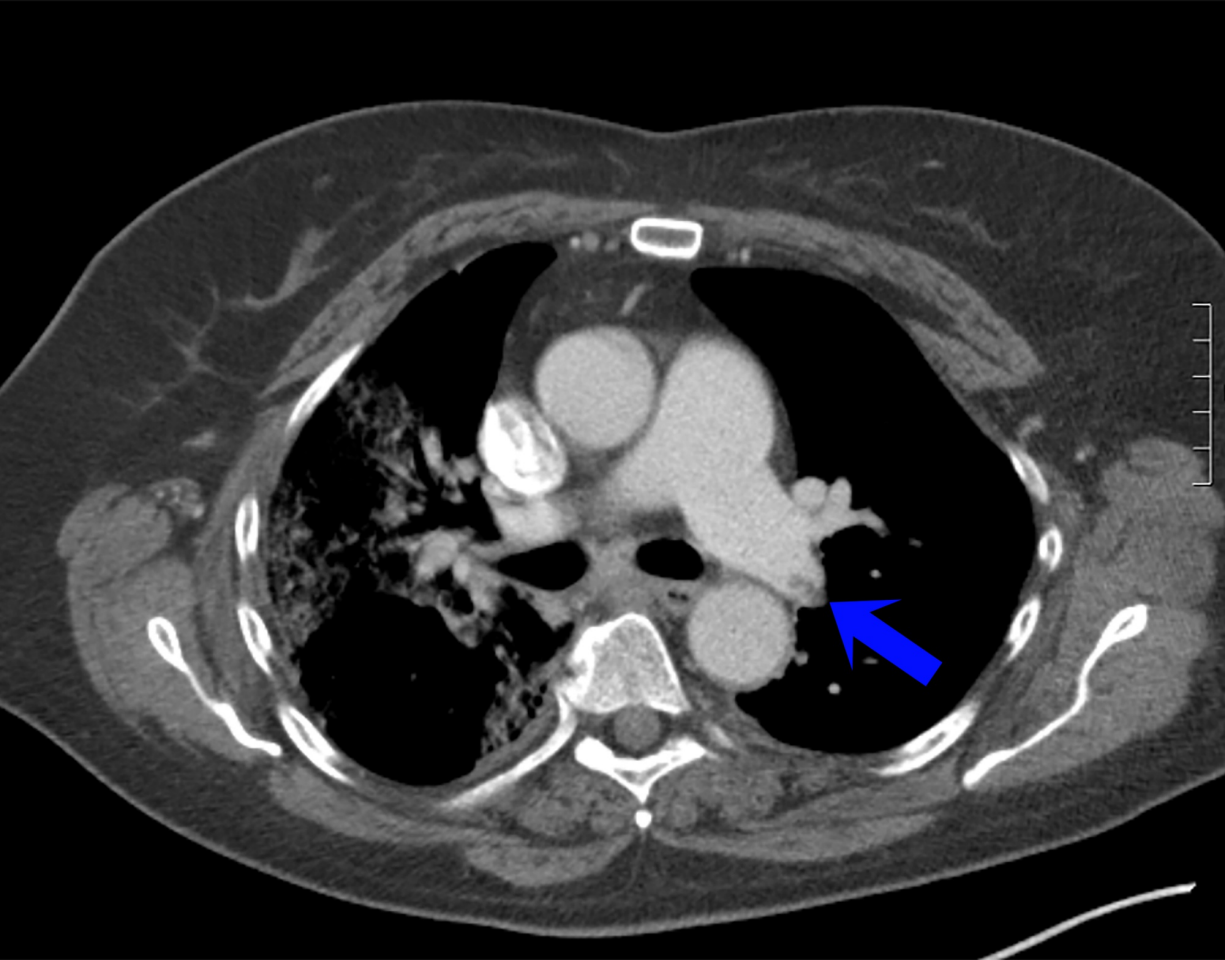
Chest CT showing bilateral ground-glass opacities with crazy paving patterns worse on the right than left, a left upper lobe pulmonary embolism with associated right heart strain, and an enlargement of the main pulmonary artery

A 64-year-old female nursing home medical technician presented to the emergency department with fever and nonproductive cough of one-week duration. Her medical history was notable for venous insufficiency, vertigo, migraine headaches, hypothyroidism, and remote tobacco abuse. In the emergency department, she was febrile (38.2℃), with an otherwise preserved heart rate, blood pressure, and respiratory rate. Her oxygen saturation was 92% on ambient air. Initial, basic laboratory diagnostics were unremarkable, and SARS-CoV2 nasopharyngeal PCR was negative. Chest radiograph demonstrated patchy infiltrates in the right mid and lower lung fields. She was discharged home with recommendations for self-isolation. Over the course of the next 48 hours, she noted ongoing fevers and worsening shortness of breath, prompting a return to the emergency department where she was noted to be febrile (39.5℃) and hypoxic (90% on room air) requiring supplemental nasal cannula. Physical examination was unremarkable except for occasional, generalized scattered crackles and wheezes on lung auscultation. Laboratory diagnostics demonstrated a preserved white blood cell count (5.8 k/uL) with neutrophilic predominance (82.6%) and lymphopenia (13.4%), with an absolute neutrophil count (4.8k/uL) to absolute lymphocyte count (0.8 k/uL) ratio of 6, a preserved hemoglobin (14.1 gm/dL), and mild thrombocytopenia (142 k/uL). The metabolic panel demonstrated mild hyponatremia (130 mmol/L), a preserved creatinine (0.90 mg/dL), and mild elevation of the AST (56 units/L) with a preserved ALT (27 units/L). Inflammatory markers were remarkable for an elevated CRP (118 mg/L), LDH (460 units/L), ferritin (1,962.5ng/mL), and D-dimer (3.38 mcg/mL). Repeat SARS-CoV2 testing on re-presentation as well as one additional test during the course of her hospitalization were negative. Computed tomography was ordered in the setting of her worsening hypoxia and elevated D-dimer and demonstrated bilateral ground-glass opacities with crazy paving patterns worse on right than left, consistent with SARS-CoV2 infection, a left upper lobe pulmonary embolism with associated right heart strain, and an enlargement of the main pulmonary artery (Figure 1). She was initiated on therapeutic anticoagulation with low molecular weight heparin (LMWH). Unfortunately, despite continued management of her pulmonary embolism and conservative management for presumed SARS-CoV2 infection, her clinical condition was complicated by progressive tachypnea and associated hypoxia, necessitating endotracheal intubation and mechanical ventilation. She received three doses of hydroxychloroquine and seven days of azithromycin. Antibiotics were discontinued as she had no evidence of secondary superinfection. Intriguingly, despite initially negative nasopharyngeal swabs, endotracheal sampling demonstrated SARS-CoV2 positivity. She remained on mechanical ventilation was subsequently extubated after seven days. She was transferred to the medical service where her clinical course remained stable and subsequently discharged home on apixaban.

## Discussion

SARS-CoV2 is an enveloped, single-stranded positive-sense RNA virus that belongs to the same class of other well-recognized coronaviruses (including SARS-CoV1, MERS-CoV) as well as those strains resulting in the common cold (HCoV-229E, HCoV-NL63, HCoV-OC43, HCoV-HKU1). To mediate its downstream effects, these positive-sense RNA-based enveloped coronaviruses must bind to cell surface receptors, undergo receptor-mediated endocytosis, and subsequent cellular entry where the virus is able to utilize host machinery to elaborate its genome. In the case of SARS-CoV1 and SARS-CoV2, full-length sequencing has demonstrated significant homology with near-identical sequences in the highly conserved spike glycoprotein (S-protein). This S-protein binds to the host receptor, angiotensin-converting enzyme 2 (ACE2), and is subsequently endocytosed in a process facilitated by host-encoded proteases - transmembrane protease serine 2 (TMPRSS2), human airway trypsin-like protease (HAT), or furins, a process exploited by multiple ACE2-binding viruses including SARS-CoV1 and Influenza [3]. Once endocytosed, progressive maturation and acidification of early endosomes and cathepsin activity facilitates viral cytosolic release and access to host cellular machinery for translation and replication.

While the clinical manifestations of SARS-CoV2 appear to be primarily mediated by direct cellular damage induced by a viral infection, however, it is becoming increasingly recognized that a variety of other signaling cascades may be responsible. SARS-CoV2 has been shown to mediate the downregulation of the ACE2 receptor, with resultant associated overactivation of the renin-angiotensin-aldosterone system (RAAS) [4]. In normal physiology, ACE1 results in the production of angiotensin II, with downstream effects of vasoconstriction, altered vascular permeability, induction of endothelial and alveolar epithelial cell apoptosis, cytokine synthesis and release, and aldosterone synthesis and release. ACE2 functions to cleave angiotensin I and angiotensin II, which not only prevents angiotensin I and angiotensin II-mediated effects, but also generates Ang1-9 and Ang1-7, respectively; peptide fragments that have inhibitory effects on vascular permeability, cardiac remodeling, and vasodilatory effects. Decreased levels of Ang1-7 are associated with increased tumor necrosis factor-alpha (TNF-α) production resulting in a pro-inflammatory state. Furthermore, RAAS-mediated signaling has been shown to result in the elaboration of inflammatory cytokines such as TNF-α and nuclear factor kappa-light-chain-enhancer of activated B cells (NFκB). The cell itself is not an innocent bystander to viral infection; it activates components of the innate immune system, such as Toll-like receptors that bind to pathogen-associated molecular patterns, allowing for antigen processing and presentation to T-lymphocytes, and inflammasome activation, with resultant downstream upregulation of a similar repertoire of inflammatory cytokines; interleukin-1 (IL-1), IL-6, TNF-α. While classic viral infection and cases of mild SARS-CoV1 and SARS-CoV2 infection seem to be typified by a limited inflammatory response, with a relative balance of pro- and anti-inflammatory signaling, severe cases of SARS-CoV2 appear to be characterized by a robust inflammatory response and associated immune dysregulation [5].

The prior coronavirus epidemics caused by SARS-CoV1 and MERS-CoV were associated with a variety of thrombotic and hematological complications. Autopsy case reports of SARS-CoV1 patients showed widespread venous and arterial thrombosis to the brain, heart, lung, renal, liver, pancreas, and extremities. A recent retrospective study on SARS-CoV2 showed that 31% of the patients developed thrombotic events (27% venous and 3.7% arterial thromboses) despite prophylactic anticoagulation [6]. Initially, COVID-19-associated coagulopathy (CAC) was described as a DIC-like consumptive coagulopathy [7], with associated thrombocytopenia, increased D-dimer (fibrinogen degradation product), and subsequently low fibrinogen. Indeed, an increased D-dimer appears to be a consistent finding not only in SARS-CoV2 infection but also in CAC, with higher D-dimer levels found in non-survivors, whereas additional DIC markers (platelet count and clotting times) demonstrated no specific association with disease. Several studies seem to suggest that patients with SARS-CoV2 infection can have mildly low, normal, or elevated platelet count. Compared to patients with non-SARS-CoV2 infection (details not specified), Yin and colleagues [8], demonstrated that patients with SARS-CoV2 infection had relative thrombocytosis, likely secondary to increased thrombopoietin, secondary to pulmonary infection/inflammation, highlighting the potential utility of platelet count as a SARS-CoV2 surrogate disease marker. Clotting times including activated partial thromboplastin time and prothrombin time were highly variable, as were levels of fibrinogen, with a trend towards more elevated levels of the latter marker. Taken together, while the D-dimer was elevated, a feature shared with DIC, the marked variability in the platelet count, clotting times, and fibrinogen levels, suggest strong dissimilarities between CAC and DIC, suggesting an alternative etiology such as inflammation-related coagulopathy. Of note, sepsis-induced coagulopathy and DIC have been reported in severely-ill COVID-19 patients and non-survivors. One study described CAC as a combination of low-grade DIC and localized pulmonary thrombotic microangiopathy, also known as pulmonary intravascular coagulopathy [9]. Interestingly, in SARS-CoV2 patients admitted with other potentially severe thromboses, such as stroke, several studies demonstrated positive laboratory markers for antiphospholipid antibody, suggesting the possibility of an underlying virally-mediated antiphospholipid antibody syndrome. Intriguingly, antiphospholipid autoantibody has been shown to transiently rise in non-SARS-CoV2 critically ill patients or those with infections and has been shown to induce endothelial dysfunction and platelet aggregation in the absence of underlying coagulopathy or rheumatological disease/ vasculitis [10].

Taken together, it appears that SARS-CoV2 is associated with an increased predisposition to a prothrombotic that is likely multifactorial in nature, involving diffuse inflammation secondary to immune system dysregulation and an ensuing cytokine storm, direct cellular injury with potential endothelial cell involvement, fibrinolysis, hypoxia-induced thrombosis, and induction of thrombosis-associated autoantibodies. Under normal physiological conditions, hemostasis is maintained by a delicate balance of pro-coagulant and anticoagulant activity with primary and secondary hemostatic events and fibrinolysis, respectively. In primary hemostasis, platelets attach to damaged endothelial cells, resulting in the formation of a platelet plug, whereas secondary hemostasis involves activation of the intrinsic or extrinsic pathways, leading to activation of the common pathway with resultant activation of thrombin and fibrin. The secondary hemostatic pathway, also known as the coagulation cascade, is regulated by numerous physiological anticoagulants - antithrombin III, tissue factor pathway inhibitor, protein C, and enzymes that trigger fibrinolysis, resulting in clot dissolution - tissue plasminogen activator and urokinase plasminogen activator. In SARS-CoV1, annexin 2, a fibrinolytic activation receptor and co-receptor of plasminogen and tissue plasminogen activator was demonstrated to have cross-reactivity with antibodies generated against SARS-CoV1, suggesting a possible autoimmune-mediated hypercoagulable state. Furthermore, these SARS-CoV1-induced annexin cross-reactive antibodies and SARS-induced cytokines were shown to be associated with dysregulation of the urokinase pathway, secondary to blockade of plasmin activity as well as up-titration of α2-plasmin inhibitor production, respectively, resulting in significant fibrinolysis, associated elevated intra-alveolar fibrin levels, and intriguingly, local thrombotic events [11].

It has been hypothesized that the SARS-CoV2-mediated dysregulation of the coagulation cascade is related to an imbalance of pro- and anti-coagulant states, which appears to be linked to the inflammatory state, cytokine activation, and cytokine storm that occurs in COVID-19 patient. SARS-CoV2 binding and endocytosis have been shown to induce expression of pro-inflammatory molecules (IL-2, IL-6, interferon-γ (IFN-γ), IFN-γ inducible protein-10 (IP-10), monocyte chemoattractant protein-1(MCP-1), macrophage inflammatory protein-1α (MIP-1α), granulocyte colony-stimulating factor (G-CSF) and TNF-α and anti-inflammatory cytokines (IL-10, TGF-beta)), suggestive of a cytokine storm. This hyperinflammatory state leads to vascular inflammation, vasomotor, and extracellular fluid dysfunction, and impairment of the coagulation pathway. Thus, cytokine storm causes disproportionate activation of endothelial cells (including direct damage) and platelets, with disruption of primary hemostasis. Endothelial cell and platelet activation trigger the release of coagulation factors including free thrombin. Furthermore, cytokine storm, a scenario characterized by excessive and unregulated immune system activation and a marked hyperinflammatory state has been associated with dysregulation of physiologic anticoagulants including antithrombin III, tissue factor pathway inhibitor, and protein C, further contributing to a prothrombotic state. This dysregulation of pro and anti-coagulation states, in part due to a tip in the relative balance of the pro-inflammatory cascade, is hypothesized to predispose to cross-linked fibrin clots, systemic infarcts, DIC, and multi-organ failure. The disproportionate disintegration of these clots, with a high burden of fibrin, leads to elevated D-dimer markers, fibrinogen level, and a prolonged PT and aPTT. Autopsy findings in severe SARS-CoV2 patients demonstrated a hypercoagulable state, revealing extensive pulmonary megakaryocyte and endothelial edema consistent with coagulation cascade activation. Ackermann and colleagues reported autopsy of seven lungs with SARS-CoV2 infection that demonstrated diffuse alveolar damage. In this autopsy analysis, the authors found perivascular T-lymphocyte infiltration, necrosis of alveolar lining cells, pneumocyte type 2 hyperplasia, and linear intra-alveolar fibrin deposition. There were associated severe endothelial injury and the presence of the intracellular (and extracellular) virus, suggesting direct viral cytopathological effects on the endothelium, with associated perivascular inflammation, disrupted cell membranes, and widespread thrombosis with microangiopathy and occlusion of alveolar capillaries. SARS-CoV2 patient samples were compared to seven historical H1N1 influenza and noted an increased prevalence of alveolar-capillary microthrombi, angiogenesis (with intussusceptive blood vessels), and thrombosis in SARS-CoV2 compared to H1N1. The study also showed significantly greater numbers of ACE2-positive cells in the lungs of patients with COVID-19 and influenza than in uninfected controls [12].

Currently, there are no definitive recommendations regarding the use of prophylactic or therapeutic anticoagulation for the management of thrombotic complications in SARS-CoV2 infection or regarding the use of D-Dimer levels to guide clinical management. Historically, D-dimer is a non-specific and an indirect marker of thrombosis, however, in the setting of suspected infection and sepsis, higher levels have been associated with increased 28-day mortality [13]. In line with the lack of specific recommendations, Fogarty and colleagues suggested that prophylactic dose low molecular weight heparin (LMWH) does not significantly impact the progressive increase in D-dimer levels observed in patients with severe COVID-19 nor the risk of thrombosis [9]. Similarly, Klok and colleagues [6], showed that thrombotic complications remain high despite the administration of thromboprophylaxis in COVID-19 patients (31% of 184 ICU patients had thrombotic complications despite receiving LMWH) Thus, higher dose, or even treatment-dose prophylaxis, has been recommended in some settings.

In a study by Tang and colleagues [14], it was observed that in SARS-CoV2 patients prophylactic treatment with LMWH (with 94% receiving enoxaparin 40-60 mg/day and 5% unfractionated heparin for 7 days) is associated with improvement in 28 mortality rates in heparinoid vs non-heparinoid users, as categorized by the sepsis-induced coagulopathy (SIC) score (Table 1). In patients with SIC >4 category, the mortality rate was found to be 40% in heparinoid users as compared to 64.2% with non-heparin users. In patients with SIC<4 category, the mortality rate was observed at 29% in heparin users, as compared to 22% with the non-heparin group. Similarly, an approximate 20% improvement in mortality rates was found, specifically in SARS-CoV2 patients on heparinoid treatment with D-dimer levels >3.0 ug/ml vs non-heparinoid group. Most patients in the study received prophylactic LMWH based on previous studies regarding lower venous thrombo-embolism prevalence in Asian populations; thus higher dose may be considered in Western populations [15]. Taken together, these findings appear to suggest that prophylactic treatment with LWMH or Heparin seem to be associated with lower mortality rates in patients at higher risk for thromboembolism, as defined by an elevated SIC score as well as those with D-Dimer levels above a certain cut-off (>3ug/mL). Nevertheless, practice guidelines remain center- and site-specific. As an example, The University of North Carolina at Chapel Hill uses a D-dimer of >2,500ng/mL as a reference value for initiation of intermediate-dosing of anticoagulation, whereas full-dose anticoagulation is used in those with a high suspicion for venous thromboembolism or repeated episodes of clotted dialysis tubing/catheters [16]. To further highlight the lack of ongoing consensus, Duke University utilizes a case-by-case method while other centers consider both the D-Dimer level (>1.5ug/mL) as well as the fibrinogen to determine anticoagulant dosing [17]. The International Society on Thrombosis and Haemostasis (ISTH) and National Institute for Public Health of the Netherlands, in contrast, recommended prophylactic-dose LMWH for all hospitalized patients with SARS-CoV2. Bikdeli’s consensus group stated that “the majority of panel members consider prophylactic dose, although a minority consider an intermediate or therapeutic dose to be reasonable,” a therapeutic strategy that has been adopted by many practitioners [18]. The Caprini, IMPROVE, and Padua models, among others, are often used for assessment of the risk of VTE. Guidelines have been developed to stratify SARS-CoV2-infected patients, highlighting the importance of different clinical outcomes, based on categorizations into DIC and SIC (ISTH, International Society on Thrombosis and Haemostasis, Table 1) [19].

**Table 1:** ISTH overt DIC and SIC scoring systems ISTH, International Society on Thrombosis and Haemostasis; DIC, disseminated intravascular coagulation; FDP, fibrin degradation products; SIC, sepsis‐induced coagulopathy; SOFA, sequential organ failure assessment; SOFA score is the sum of four items (respiratory SOFA, cardiovascular SOFA, hepatic SOFA, renal SOFA).

Item	Score	DIC Range	SIC Range
Platelet count (-10^9/L)	2	<50	<100
	1	>50, <100	>100, 150
FDP/D-dimer	3	Strong increase	-
	2	Moderate increase	-
Prothrombin time (PT ratio)	2	<6s	1.4
	1	>3s, <6s	(1.2, <1.4)
Fibrinogen (g/mL)	1	<100	-
SOFA score	2	-	>2
	1	-	1
Total score for DIC or SIC		>5	>4

The ISTH recommended extended prophylaxis with LMWH or direct oral anticoagulants up to 45 days after discharge for those with elevated VTE risk (such as those with reduced mobility or active cancer) and with elevated D-dimer more than twice the upper limit of normal, though the latter recommendation failed to achieve unanimous approval [18]. Despite the initiation of anticoagulation, continued evaluation for the development of de novo thrombosis is recommended [20].

## Conclusions

In SARS-CoV2 patients, the risk of thrombosis appears to be multifactorial - direct viral cytopathological effects, a pro-inflammatory state, cytokine storm, hypoxia-inducible thrombosis, and endothelial inflammation culminating in the formation of intra-alveolar or systemic fibrin clots. While initial guidelines have been developed to assist clinicians in selecting appropriate chemoprophylaxis as well as therapeutic anticoagulation, consensus statement remain lacking. Further studies are needed to evaluate the pathogenesis of SARS-CoV2.
